# Risk of lingual nerve injuries in removal of mandibular third molars: a retrospective case-control study

**DOI:** 10.1186/s40902-019-0222-4

**Published:** 2019-09-10

**Authors:** Itaru Tojyo, Takashi Nakanishi, Yukari Shintani, Kenjiro Okamoto, Yukihiro Hiraishi, Shigeyuki Fujita

**Affiliations:** 1 Department of Oral and Maxillofacial Surgery, Wakayama Medical University, 811-1 Kimiidera, Wakayama, Wakayama 641-8509 Japan; 2 Department of Dentistry and Oral Surgery, Japanese Red Cross Wakayama Medical Center, 4-20 Komatsubara-dori, Wakayama, Wakayama 640-8558 Japan

**Keywords:** Lingual nerve injury, Mandibular third molar, Extraction, Orthopantomograph

## Abstract

**Background:**

Through the analysis of clinical data, we attempted to investigate the etiology and determine the risk of severe iatrogenic lingual nerve injuries in the removal of the mandibular third molar.

**Methods:**

A retrospective chart review was performed for patients who had undergone microsurgical repair of lingual nerve injuries. The following data were collected and analyzed: patient sex, age, nerve injury side, type of impaction (Winter’s classification, Pell and Gregory’s classification). Ratios for the respective lingual nerve injury group data were compared with the ratios of the respective data for the control group, which consisted of data collected from the literature. The data for the control group included previous patients that encountered various complications during the removal of the mandibular third molar.

**Results:**

The lingual nerve injury group consisted of 24 males and 58 females. The rate of female patients with iatrogenic lingual nerve injuries was significantly higher than the control groups. Ages ranged from 15 to 67 years, with a mean age of 36.5 years old. Lingual nerve injury was significantly higher in the patient versus the control groups in age. The lingual nerve injury was on the right side in 46 and on the left side in 36 patients. There was no significant difference for the injury side. The distoangular and horizontal ratios were the highest in our lingual nerve injury group. The distoangular impaction rate in our lingual nerve injury group was significantly higher than the rate for the control groups.

**Conclusion:**

Distoangular impaction of the mandibular third molar in female patients in their 30s, 40s, and 50s may be a higher risk factor of severe lingual nerve injury in the removal of mandibular third molars.

## Background

Injury of the lingual nerve can occur from a wide variety of oral and maxillofacial trauma, oral cancer, or other diseases and surgical procedures. The most common cause of lingual nerve injury is the removal of the mandibular third molars. Behnia et al. [1] examined 669 lingual nerves in cadavers, and found that 94 (14.05%) were above the lingual crest, and one (0.15%) was located in the retromolar pad just on the surface of the mandible. In the remaining 574 cases (85.80%), the nerve was situated in its typical position. The mean horizontal and vertical distances of the nerve from the lingual plate and the lingual crest were 2.06 ± 1.10 mm (range, 0.00 to 3.20 mm) and 3.01 ± 0.42 mm (range, 1.70 to 4.00 mm), respectively. In 26% of the cases, the nerve was in direct contact with the lingual plate of the alveolar process.

Lingual nerve injury is an uncommon but important complication in the removal of the mandibular third molar. Renton et al. reported that the incidence of lingual nerve injury was estimated to vary from 0.02 to 2% of the patients undergoing third molar surgery [2]. Pippi et al. reviewed the incidence of temporary lingual nerve injury and estimated it to vary from 0 to 37.5% of the patients undergoing third molar surgery, while the incidence of permanent lingual nerve surgery was estimated to vary from 0 to 2% [3]. Thus, the ratio of permanent lingual nerve injury is very rare.

In cases of inferior alveolar nerve injury, healing and recovery are relatively quick, as the nerve runs within the bony canal. However, the injured lingual nerve is usually not supported by a bony canal and thus, the regenerating nerve fiber tends to randomly expand within the soft tissue or scar tissue. Therefore, recovery of an injured lingual nerve is relatively slower than that for the inferior alveolar nerve [4, 5].

Orthopantomography or computed tomography can be used to assess the position of the inferior alveolar canal before removal of the mandibular third molar. However, it is difficult to assess the position of the lingual nerve when using these techniques. Although Miloro et al. [6] have reported on the efficacy of using magnetic resonance imaging (MRI) to assess the lingual nerve in the third molar region, it is difficult for dental practitioners to routinely use MRI prior to the removal of the mandibular third molar.

Studies that have examined the causes of the lingual nerve damage include investigations of the method of tooth extraction, the skill of the practiced hand, and the displacement of the anatomical position of the lingual nerve, among others [2, 3, 5, 7–11]. However, detailed reports on patients with severe lingual nerve injuries needed surgical nerve restoration are rare. The aim of this current retrospective case-control study was to clarify the risks of severe iatrogenic lingual nerve injuries in the removal of the mandibular third molars.

## Methods

This retrospective clinical study examined 79 patients between March 2003 and November 2016 who had undergone microsurgical repair of their lingual nerve in the removal of their mandibular third molars at the Department of Oral and Maxillofacial Surgery, Wakayama Medical University. This study followed the Declaration of Helsinki on medical protocol and ethics, and the regional ethical review board of Wakayama Medical University approved the study.

All cases of lingual nerve injury were caused in other facilities. The criteria for performing repairs of the lingual nerve via microneurosurgery included (1) a witnessed transection, (2) two-point discrimination (2-PD) > 20 mm in the affected area over 3 months after the injury, (3) no sensation observed during a temperature test in the affected area at over 3 months after the injury, (4) no sensation observed during a taste test in the affected area at over 3 months after the injury, (5) no sensation observed during a pin-prick test in the affected area at over 3 months after the injury, and (6) finding a difference for the Semmes-Weinstein monofilament test (SWM test) between the affected and non-affected side at over 3 months after the injury. Microneurosurgery of the lingual nerve repair was indicated if (1) or all of (2–6) were present [12]. About the surgical procedure [12], the lingual nerve was exposed through an intraoral mucosal incision and lingual flap reflection. Optical magnifying glasses (250 mm) and an operating microscope (Superlux 301, Zeiss, Jena, Germany) were available during surgery. In all cases, the lingual nerves were completely disrupted and heavily trapped by dense scar tissue. Most cases showed neuromas at the torn nerve ends. The neuromas and peripheral scars surrounding the torn nerves were completely removed; after this procedure, the two nerve ends could touch without tension. As much scar tissue as possible was removed from the torn nerve, and the transected lingual nerve stumps were identified, mobilized, and trimmed to the point where the fascicles could be identified in the microsurgical field. In all cases, direct end-to-end epineural nerve sutures without tension were performed at eight or more sites around the stump, using 8-0 or 9-0 nylon. Nerve grafts were not required in any case.

**Fig. 1:**
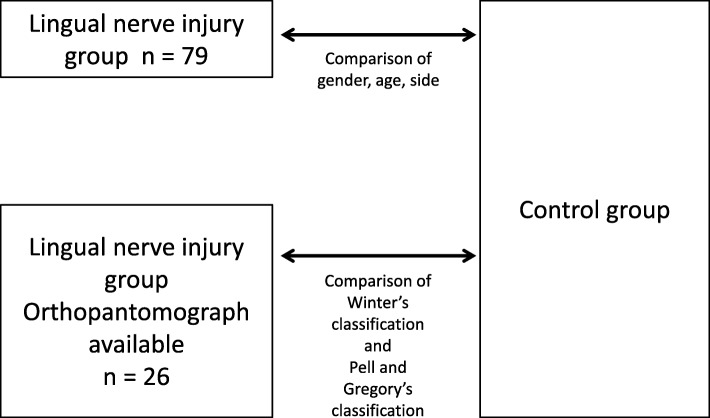
A work-flow diagram

The following data were collected and analyzed: sex, age, nerve injury side. The orthopantomographs of 26 cases were available and divided according to Winter’s classification, and the Pell and Gregory classification (Fig. 1) [13, 14].

The inclination of the longitudinal axis of the third molar was divided into distoangular, horizontal, mesioangular, vertical, inverted, buccoangular, and linguoangular based on Winter’s classification. Obtainment of the angle between the occlusal plane or line parallel to it and the longitudinal axis of the impacted third molar, in turn, allowed objective classification of the third molars according to the Winter classification. The subclasses used were as follows: (1) third molars with negative angles (< 0°) were considered to be inverted, (2) third molars with an angle between 0° and 30° were considered to be horizontal, (3) third molars with an angle between 31° and 60° were considered to be mesioangular, (4) third molars with an angle between 61° and 90° were considered to be vertical, and (5) third molars with an angle > 90° were considered to be distoangular [15].

The depth of the impacted third molar with the occlusal plane and the available space with respect to the ascending mandibular ramus were divided into positions A, B, and C, and into classes I, II, and III according to the Pell and Gregory classification.

We compared the ratios of the respective data in our lingual nerve injury group (LNIG) to the ratios of the respective data in the past literature (Fig. 1), and which was used as the control group (CG) [9, 11, 15–18]. This data came from studies that examined the various complications encountered during the removal of the mandibular third molar.

### Statistical analysis

Statistical analyses of the ratio differences between each parameter of two groups, namely lingual nerve injury group and control group, were performed using Software JMP® Pro version 12.2.0 (SAS Institute Inc., Cary, NC). Chi-squared test and *t* test were used for these analyses, with *P* < 0.05 designated as being significant.

## Results

The patient group consisted of 23 males and 56 females, with an average age of 36.5 years old. The ratio for the female patients with iatrogenic lingual nerve injuries was significantly higher than the ratios found for the female patients in the various control groups (Table 1).

**Table 1:** Comparison of LNIG and CG on gender

	This study (LNIG)	Almendros-Marqués et al, 2006 (CG) [15]	Uematsu et al, 2015 (CG) [16]	Cheng et al, 2010 (CG) [9]	Blondeau et al, 2007 (CG) [17]	Smith et al, 2013 (CG) [11]
Male (%)	27.8	47.3	41.1	39	41.6	41.5
Female (%)	72.1	52.7	58.9	61	58.4	58.5
Sample size (patients)	79	165	461	3595	327	1000
Power		0.86	0.68	0.59	0.67	0.74
*P* value		0.001	0.0294	0.07	0.0235	0.024

Abbreviations: *LNIG* lingual nerve injury group, *CG* control group

*P* value (chi-squared test): The sex ratio in this study was statistically compared to the sex ratio of the other past studies. *P* < 0.05 was considered to be statistically significant

**Fig. 2:**
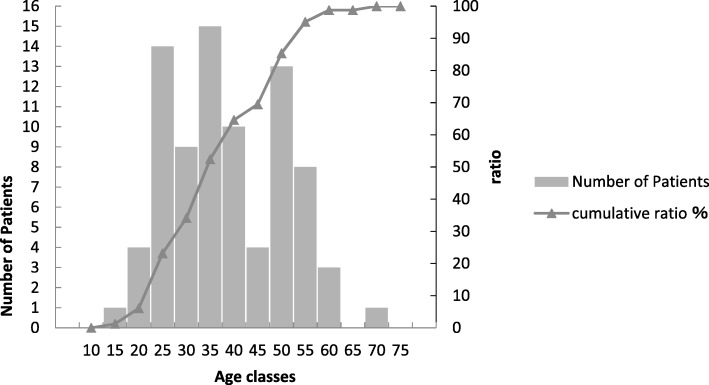
Age distribution of 79 consecutive patients undergoing microsurgical repair of the lingual nerve injuries after the removal of the mandibular third molars

Ages ranged from 15 to 67 years, with a mean age of 36.5 years old (Fig. 2). There was a significantly higher average age for the iatrogenic lingual nerve injury group versus the various control groups (Table 2).

**Table 2:** Comparison of LNIG and CG on age

	This study (LNIG)	Almendros-Marqués et al, 2006 (CG) [15]	Smith et al, 2013 (CG) [11]	Cheng et al, 2010 (CG) [9]	Uematsu et al, 2015 (CG) [16]
Age range	15-67	16-64	13-87	14-82	
Mean	36.5	27.32	33.9	27.2	30.5
Sample size (patients)	79	165	1000	3595	461
Power		0.99	0.46	1.00	0.97
P value		< 0.0001	0.0619	< 0.0001	< 0.0001

Abbreviations: *LNIG* lingual nerve injury group, *CG* control group

*P* value (t test): The mean age in this study was statistically compared to the mean age of the other past studies. *P* < 0.05 was considered to be statistically significant

The side of the lingual nerve injury was located on the right in 44 and on the left in 35 patients. There was no significant difference between the ratio of the lingual nerve injury side and the ratio for the removal side for the mandibular third molar in the control group (Table 3).

**Table 3:** Comparison of LNIG and CG on extraction side of mandibular third molar

	This study (LNIG)	Uematsu et al, 2015 (CG) [16]
Right (%)	55.7	49.7
Left (%)	44.3	50.3
P value		0.28

Abbreviations: *LNIG* lingual nerve injury group, *CG* control group

*P* value (chi-squared test): The ratio of the lingual nerve injury side in this study was statistically compared to the ratio of the mandibular third molar removal side in the other past study. *P* < 0.05 was considered to be statistically significant

With respect to inclination of the longitudinal axis of the mandibular third molars, the ratios were 30.8% for distoangular, 30.8% for horizontal, 19.2% mesioangular, 11.5% for vertical, 3.8% for inverted, 3.8% for linguoangular, and 0% for buccoangular. With respect to the depth of the mandibular third molars, the ratios were 26.9% for position A, 69.2% for position B, and 3.8% for position C. The ratios for the available space from the mandibular second molar to mandibular ramus were 23.1% for class I, 61.5% for class II, and 15.4% for class III. The highest ratios in our lingual nerve injury group were found for the distoangular and horizontal (Table 4). There was a significantly higher ratio for the distoangular impaction in the lingual nerve injury group compared to the ratio for the general distoangular impaction type in the control group (Table 5).

**Table 4:** Classification of mandibular third molar based on the Pell and Gregory and the Winter’s criteria

Classification	Number	%
Inclination of the longitudinal axis of the molar		
Distoangular	8	30.8
Horizontal	8	30.8
Mesioangular	5	19.2
Vertical	3	11.5
Inverted	1	3.8
Linguoangular	1	3.8
Buccoangular	0	0
Depth (with respect to occlusal plane)		
Position A	7	26.9
Position B	18	69.2
Position C	1	3.8
Available space (with respect to ascending mandibular ramus)		
Class I	6	23.1
Class II	16	61.5
Class III	4	15.4
IA	1	3.8
IIA	6	23.1
IIIA	0	0
IB	5	19.2
IIB	9	34.6
IIIB	4	15.4
IC	0	0
IIC	1	3.8
IIIC	0	0

The twenty six cases evaluated by orthopantomography were divided according to the Winter’s classification and the Pell and Gregory classification

**Table 5:** Comparison of LNIG and CG based on Winter’s classification

	This study (LNIG)	Cheung et al, 2010 (CG) [9]	Almendros-Marqués et al, 2006 (CG) [15]	Uematsu et al, 2015 (CG) [16]	Oguma et al, 2013 (CG) [18]	Smith et al, 2013 (CG) [11]
Distoangular (%)	30.8	10.7	15.8	1.2	0.2	31.0
Horizontal (%)	30.8	26.0	12.4	50.9	66.7	13.0
Mesioangular (%)	19.2	47.9	20.5	27.5	18.7	32.0
Vertical (%)	11.5	15.4	47.9	19.7	13.9	21.0
Inverted (%)	3.8	0	3.5	0.7	0.5	0
Other (%)	3.8	0	0	0	0	3.0
*P* value (disto-)		0.009	0.036	<0.0001	<0.0001	0.979
*P* value (horizo-)		0.437	0.023	0.991	1	0.029
*P* value (mesio-)		0.997	0.755	0.929	0.682	0.973
*P* value (vert-)		0.738	1	0.879	0.668	0.906
*P* value (invert-)			0.575	0.155	0.002	

Abbreviations: *LNIG* lingual nerve injury group, *CG* control group

*P* value (chi-squared test): The ratio of Winter's classification in this study was statistically compared to the ratio found in other past studies. *P* < 0.05 was considered to be statistically significant

## Discussion

Cheung et al. [9] reported that the distoangular impaction significantly increased the risk of the LN deficit (*P* < 0.001). They additionally reported that the lingual nerve deficit according to the type of impaction ranged from 0.53% each for the mesioangular and horizontal to 2% for the distoangular. This variation in the incidence according to the impaction type was statistically significant. Similarly, Juodzbalys et al. reported that the incidence of lingual nerve injury was highest for the distally impacted lower wisdom teeth (4.0%, *P* < 0.01), followed by horizontal impaction (2.8%), mesial impaction (2.4%), and vertical impaction (1.9%) [10]. However, Jerjes et al. reported that there was a much higher prevalence of permanent lingual nerve paresthesia in the group of patients with horizontally impacted third molars (6.3%), with the other risk factors of lingual nerve injury including male patients, close radiographic proximity to the inferior alveolar canal, and treatment by trainee surgeons [5]. Similar to the findings of the Cheung et al. study, our data also showed that the removal of the distoangular mandibular third molars was strongly associated with lingual nerve injuries. However, there could be racial differences between the Cheung et al. and Jerjes et al. data that need to be taken into consideration.

**Fig. 3:**
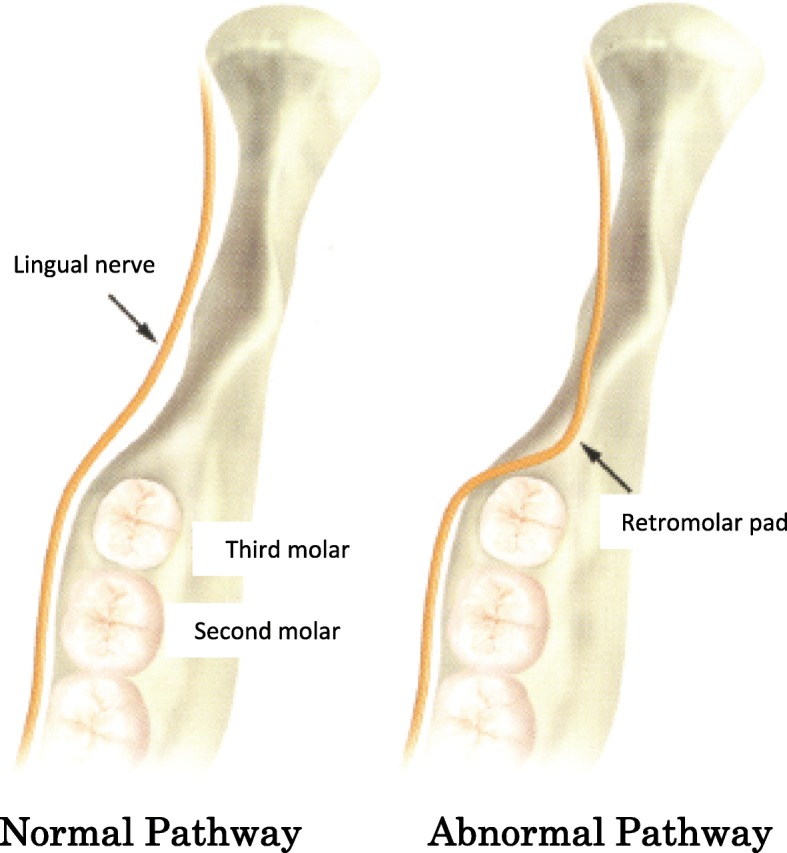
Lingual nerve pathway and distoangular mandibular third molar position. Arrows indicate the distoangular directions

The reasons suggested for the high ratio of lingual nerve damage for the distoangular position during the removal of the mandibular third molar may be as follows. It is possible that the tooth crown could be anatomically close to the region of the lingual nerve (Fig. 3). Thus, the distal and lingual sides have a greater exposure when the tooth is dislocated at the time of the tooth extraction. As a result, the lingual nerve is more likely to be injured when the bone of the distal region of the tooth is shaved. Distolingual bones of mandibular third molars were shaved in five out of the eight distoangular mandibular third molar cases in our study. Valmaseda-Castellón et al. reported that third molars with lingual angulation were associated with the occurrence of lingual nerve injury (Fisher exact test: *P* = .09; odds ratio = 4.39) [8]. Anatomically, the distoangular mandibular third molar involves lingual angulation. This is because the lingual plate and nerve are located in the distal position of the mandibular third molar, with the lingual bone shape of the transition region from mandibular body part to ramus. During the removal of the distoangular or lingual inclination mandibular third molars, the shaving of the pericoronal bone of the lingual or distolingual site was shown to be strongly associated with lingual nerve injury.

Cheung et al. reported that 0.91% (13/1427) of the procedures that involved a raised lingual flap resulted in postoperative lingual nerve deficits, while there were only 0.58% (17/2911) extraction cases with a postoperative deficit when the lingual flap was not raised, although there was no significant difference between these two proportions (*P* = 0.58) [9]. In our study, however, it was unknown if the lingual flap was raised or if the lingual retractor was inserted under the lingual flap.

With respect to sex, our study indicated that there was a significantly higher ratio of female iatrogenic lingual nerve injury patients versus the control groups. Jaw and mouth opening sizes of the patients may be involved with iatrogenic lingual nerve injury. However, Cheung et al. reported finding no association between a patient’s sex and the risk of inferior alveolar nerve and lingual nerve deficits [9]. Jerjes et al. reported that there was significantly higher permanent lingual nerve paresthesia (*P* = 0.002) in male patients [5].

With respect to age, our study showed there was a significantly higher average age for the iatrogenic lingual nerve injury group compared to the various control groups. Bone hardness of the mandible due to aging may also be involved with iatrogenic lingual nerve injury. Chiapasco et al. and To et al. both reported finding a relationship between increasing age and an increasing risk of lingual nerve injury [19, 20]. However, the findings of the Cheung et al.’s study did not support the hypothesis that there was an age-associated increased risk of inferior alveolar nerve and lingual nerve deficits due to lower third molar surgery [9]. Furthermore, Valmaseda-Castellón et al. also found that there was no association between either the age or sex and the lingual nerve [8].

Although Renton et al. have reported that the incidence of lingual nerve injury is estimated to vary from 0.02 to 2% of patients undergoing third molar surgery [2], the findings by Fielding et al. [21] suggested that the incidence of lingual nerve damage following third molar surgery was more frequent than once thought. Fielding’s study sent 600 questionnaires to fellows of the American Association of Oral and Maxillofacial Surgeons. Of the 452 respondents, 76.5% reported having had patients with lingual anesthesia, dysesthesia, or paresthesia. Of all the reported cases, 18.64% failed to resolve. Therefore, it is very important that lingual nerve injury should be prevented during third molar surgery. The results of our study suggest that orthopantomography evaluation prior to mandibular third molar extraction can be performed to determine the type of the mandibular third molar that is the present, with the findings then used to determine the risk of severe lingual nerve injury. However, in order to compare our findings with severe lingual nerve injury data, we used mandibular third molar removal data from the literature, which included heterogeneous elements as the control group. For a more accurate evaluation of the risk factor for lingual nerve injury during the removal of the mandibular third molar, long-term multi-institutional prospective research will need to be undertaken, as the incidence rate of lingual nerve injury during the removal of the mandibular third molar is rare.

## Conclusion

Distoangular impaction was found to significantly increase the risk of severe lingual nerve injury in the removal of the mandibular third molars. Female patients and age may be associated with lingual nerve injury in the removal of the mandibular third molar.

## Data Availability

Please contact the author for data requests.

## Abbreviations

CG: Control group
LNIG: Lingual nerve injury group
MRI: Magnetic resonance imaging
